# Organisational drivers of performance in mental health providers

**DOI:** 10.1108/JHOM-01-2022-0017

**Published:** 2023-03-17

**Authors:** Russell Mannion, Frederick Hassan Konteh, Rowena Jacobs

**Affiliations:** Health Services Management Centre , University of Birmingham , Birmingham, UK; Centre for Health Economics , University of York , Heslington, UK

**Keywords:** Mental health providers, Governance, Organizational culture, Performance, English NHS

## Abstract

**Purpose:**

This study aims to compare and contrast the core organisational processes across high and low performing mental health providers in the English National Health Service (NHS).

**Design/methodology/approach:**

A multiple case study qualitative design incorporating a full sample of low and high performing mental health providers.

**Findings:**

This study suggests that the organisational approaches used to govern and manage mental health providers are associated with their performance, and the study’s findings give clues as to what areas might need attention. They include, but are not limited to: developing appropriate governance frameworks and organisational cultures, ensuring that staff across the organisation feel “psychologically safe” and able to speak up when they see things that are going wrong; a focus on enhancing quality of services rather than prioritising cost-reduction; investing in new technology and digital applications; and nurturing positive inter-organisational relationships across the local health economy.

**Originality/value:**

Highlights considerable divergence in organisation and management practices that are associated with the performance of mental health trusts in the English NHS

## Introduction

Advances in the science of health informatics have helped to expose wide and persistent variations in the performance of health care providers. The presence of such variation raises difficult questions about equity, quality and consistency of care and suggests potential opportunities for learning and improvement. Notwithstanding the limitations that bedevil healthcare performance metrics, or maybe also for this reason, observed variations in performance have stimulated a keen interest amongst academics and policy makers alike in understanding why some health care organisations perform better than others. This is because increasing attention is being devoted to the creation of explicit and finely tuned financial incentives in the form of payment for performance programmes. The literature on the drivers of provider performance in health care is vast and offers a rich inventory of explanations for observed differences. Factors external to providers that have been investigated empirically include, the degree of market competition ( Bijlsma *et al* ., 2013 ; Longo *et al* ., 2017 ; Cooper *et al.* , 2016 ) accreditation and inspection systems ( Hussein *et al.* , 2021 ; Brubakk *et al.* , 2015 ); and local labour markets ( Burgess *et al* ., 2003 ).

Since the publication in the USA of the Institute of Medicine's landmark report *Crossing the Quality Chasm* ( IOM, 2001 ) and in the UK the equally pivotal *An Organisation With A Memory* ( Department of Health, 2000 ) – both of which identified organisational systems and cultures as key sources of underlying problems of quality, there has been growing interest in exploring management and organisational factors as possible explanations for variations in the performance of health care organisations ( West *et al* ., 2002 ; Ramanujam and Rousseau, 2006 ).

### Studying organisational factors and performance in health care organisations

Empirical research investigating the organisational drivers of performance can be classified into one of five broad categories ( Lega *et al* ., 2013 ; Chambers *et al* ., 2013 ; Mannion *et al* ., 2016 ). First, those studies that explore the impact of *management practices* on performance. For example, Bloom and colleagues, gathered data on management styles and practices across more than 2,000 hospitals in the UK, the USA, Canada, France, Germany, Sweden, Italy, Brazil and India. They found that hospitals with higher management-practice scores (assessed by a standardised questionnaire) with regard to planning, organising, coordinating and controlling, had better clinical outcomes and stronger financial performance ( Bloom *et al* ., 2014 ). Second, a stream of work that has investigated the impact of *managers' characteristics* on performance. Third, empirical research which examines the impact of *professional engagement* in organisational performance management. For example, in a national quantitative study in the English National Health Service (NHS) it was found that increased clinical involvement, particularly the number of doctors on acute hospitals (NHS Trusts) boards is linked to better organisational performance as measured by external regulator quality ratings ( Veronesi *et al* ., 2013 ). Fourth, studies which explore the link between leadership competencies and performance. For example, Mannion *et al* . (2017) in a national quantitative study exploring the relationship between hospital board competencies and a range of process and outcome indicators across all hospital trusts in the NHS, found that board member competencies were linked to the willingness of staff to speak up about threats to patient safety and perceptions that senior managers would respond appropriately to such concerns when they were raised. Finally, a strand of research which investigates the relationship between *organisational cultures and performance* . For example, Jacobs *et al* . (2013) in a national quantitative study examined the relationship between senior management team culture and organisational performance in English hospital trusts using a validated culture rating instrument, the competing values framework (CVF). The study found evidence to suggest a strong relationship between dominant cultural type and a variety of routine measures of organisational performance.

Yet, the organisational factors that drive performance in complex, volatile and high stakes health care environments characterised by multiple competing objectives and diffused authority structures are difficult to isolate and disentangle with quantitative approaches alone. This is particularly the case when correlational analysis is based on cross sectional designs, which at best can only reveal associations between variables but cannot establish true causality. In this situation in-depth qualitative case studies can be used to augment quantitative approaches by generating rich contextual knowledge of the inner workings of health care organisations and thereby provide more nuanced explanations of how and why some providers are able to achieve better performance than others. Of relevance here is a small, but growing body of research based on positive deviance methodology. This approach combines the benefits of both quantitative and qualitative methods and has been used to generate insights useful for explaining variance in health care provider performance ( Baxter *et al.* , 2016 ). The sequential steps in this approach involve identifying “positive deviants,”–those organisations with uncommon but successful practices that lead to higher performance than their peers. These are then subject to in-depth qualitative investigation to explore their internal organisational drivers of performance ( Bradley *et al.* , 2009 ). Positive deviance methods are particularly appropriate in situations where providers can be ranked reliably based on robust performance measures and where there is substantial natural variation in performance amongst providers ( Baxter *et al* ., 2019 ). However, it may be the case that exceptional performance is achieved through non-deviant means and in such situations a complementary design may be required, such as studies based on a detailed comparison of the internal organisational characteristics of high and low performing organisations. Such an approach is based on the assumption that sampling organisations at either end of the performance spectrum is likely to offer sharper contrasts and more useful insights than one based on an undifferentiated sample of high or middling performers. For example, drawing on a similar research design, Mannion *et al* . (2005b) used a multiple case study design which incorporated a purposeful sample of low and high performing acute hospitals in the English NHS to explore how they differed in terms of their dominant cultural attributes and how these cultural attributes influenced their performance in positive and negative ways.

### Aim of the study

Against this theoretical and empirical background, we sought empirical substantiation to discern whether there is a patterning in the configuration of core organisational processes and management practices across high and low performing mental health trusts.

## Methodology

### Institutional context

This study was conducted within the English NHS–a publicly funded, single-payer health care system that provides universal coverage with most services provided free at the point of service. Mental ill health is the single largest cause of disability in the UK, contributing up to 23% of the total burden, compared to 16% for cancer and 16% for cardiovascular disease, but mental health services receive only 13% of the total NHS budget ( All Parliamentary Committee, 2015 ). There are 54 mental health trusts in England which provide a range of health and social care support services for people living with a mental health illness. The services provided by mental health trusts range from psychological or talk therapy to highly specialised care for people with severe mental health problems. Mental health trust services are commissioned and funded by clinical commissioning groups – groups of general practitioners and other health professionals who purchase services on behalf of their local population based on need. Access to secondary mental health services is usually arranged through the patient's primary care medical doctor or sometimes via self-referral or the criminal justice system. Most services are for those who live in the region, although some mental health trusts may accept national referrals. Improving the performance and efficiency in mental health services has long been a national policy priority and recent years have witnessed substantial reform in the way mental health trusts are reimbursed. Yet, and unlike the acute sector, there is a dearth of evidence on the underlying drivers of cost, quality and performance in mental health services.

#### Study design, sampling and data collection

We utilised a comparative case-study design to explore the interplay between organisational factors and performance in four purposefully sampled mental health trusts, comprising:

1. two trusts that the data suggest provide low cost/high quality care;
2. two trusts that the data suggest provide high cost/low quality care

The selection of the four trusts used a three-stage process. First, purposeful sampling of two low and two high performing mental health trusts using a novel method for assessing performance. This involved applying estimates from a discrete choice experiment eliciting the UK general population as well as service user and mental healthcare professionals' valuation of quality attributes, to administrative and patient survey data, alongside measures of provider costs, to quantify the relative performance of trusts in terms of cost and quality and compare rankings of providers (See Rowen *et al* ., 2022 ; Aragón *et al* ., 2022 for more details). Second, we selected trusts in each performance category, which had similar external factors believed to affect performance: local population characteristics, rurality, size and levels of competition. The rationale was to enable the attribution of differences in performance to internal organisational processes, rather than external factors, over which trusts have limited control. Finally, we ensured that the final sample of trusts contained those that served a predominantly rural population as well as those that served a predominantly urban population. We contacted four trusts, which met these criteria and three agreed to participate. We then contacted a fifth trust which met the criteria. This trust agreed to participate and replaced the original selection. The sites have been renamed A-D to protect anonymity.

*Trust A* was assessed as a low performing trust. It provides the full range of mental health services and support for people with mental illness and learning disabilities and employs over 2,400 staff. It is based in the East Midlands area of England in a rural setting and serves a population of more than 700,000 people.

*Trust B* was assessed as a low performing trust. It provides a range of community, mental health and acute hospital services and employs over 4,000 staff. It is based in the South West of England in a mostly rural area with no large urban centre.

*Trust C* was assessed as a high performing trust providing a range of integrated mental health and social care services to people of all ages and employs more than 2,000 staff. It is based in a major city in England and serves a population of over 1.2 million people

*Trust D* was assessed as a high performing trust. It provides specialist mental health, learning disability and community health services and employs over 3,500 staff. It is based across a mixed urban setting and serves a population of more than 900,000 people.

The primary mode of data collection involved semi structured interviews. We undertook 60 interviews across the case study sites, between November 2019 and August 2021. Participants were purposefully selected in each site to include senior managers and clinicians with a knowledge of strategic service planning, patient representatives and senior managers from clinical commissioning groups (CCG) that commission services from the provider, to provide an external perspective. Across the sites these included trust chief executives (three); medical/clinical directors (four); directors of nursing (three); other board directors (including Chair, Finance, Strategy, Communications and Operations) (12); service managers with a range of organisational roles and responsibilities (24); consultant psychiatrists (four); senior managers from local clinical commissioning groups (six); and patient representatives (four). Table 1 details the roles of interviewees across the four sites.

Interviews were conducted face-to-face and using online video conferencing software and lasted between 45 and 60 min. With the consent of participants, interviews were digitally recorded and professionally transcribed verbatim. Information from interviews was supplemented with field notes. A topic guide based on themes derived from the relevant theoretical and empirical literature, as outlined earlier, was used to structure the interviews and centred on the following:

1. Board *governance* : style of leadership and the strategic priorities of the trust board.
2. Organisational *culture* : values underpinning working practices and in particular how open the organisation is to hearing and acting on the concerns of staff
3. Organisational *relationships* : with the local health economy
4. Organisational *technology* : information and communication systems, including digital platforms for delivering and accessing care.

The preceding topics were departures for discussions rather than a fixed schedule. Interviews were allowed to proceed as conversations, and new themes were taken up and explored as these arose. Therefore, we adopted an abductive research approach, enabling the application of both deductive and inductive reasoning to our enquiry ( Awuzie and McDermott, 2017 ).

### Data analysis and interpretation

Qualitative coding software (NVivo) was used to facilitate data storage and retrieval in analysis. The five stages of the framework method (familiarisation, theme identification, indexing, charting and interpretation) were followed and structured the analysis of data ( Gale *et al* ., 2013 ). In order to improve the validity of the study, where possible, we cross-referenced accounts from individuals and triangulated the evidence emanating from different sources, including internal documents (e.g. clinical governance reports) and external reports (published CQC assessments). We also audited the various sources of data in order to search for negative or disconfirming evidence that appeared to contradict or was inconsistent with the emerging analysis. The coded material was discussed during analysis meetings involving all members of the research team. Themes and data were scrutinised and compared to pinpoint similarities, differences and refine themes. Analysis was initially conducted at a “within-case” level to integrate and triangulate data in order to holistically describe the relationship between internal processes and organisational performance. Cross-case comparisons were then conducted across the sites to identify important similarities and differences. While there is a patterning of experience which is unique to each case study site, our analysis and presentation of findings extend their individual value by integrating the common themes across the four sites and in particular drawing out key differences between the two low and two high performing organisations.

## Results

The key points of divergence in organisational and management practices identified can be grouped under four broad headings, each of which is discussed subsequently (see Table 2 ):

### Board leadership and governance

Mental health trusts in the English NHS are derived in structure from the Anglo-Saxon private sector unitary board model. The unitary board typically comprises a chairperson, chief executive, executive directors and governors comprising elected people from the local community. Boards are collectively responsible for all aspects of the operation and performance of the trust and have a statutory responsibility for upholding of quality and safety of care in their organisation. The executive directors each has particular responsibility for leading a specific function or service domain. Non-executive directors do not have formal managerial roles, but are responsible for challenging the executive directors in decision-making and on the trust's strategy. In addition, freestanding foundation trusts have a board of governors drawn from the local community to provide additional governance mechanisms attuned to local needs.

We found little difference between the two high and the two low performing trusts with regard to the composition of their boards and the delegated roles and tasks undertaken by executive and non-executive members. This is perhaps an unsurprising finding given that the role and functions of trust boards are prescribed in legislation, albeit with some discretion for local differences in the membership of boards. However, we did uncover evidence to suggest there is a relationship between the leadership style of the trust board and organisational performance. Broadly, we found that the two low performing trusts were characterised by a top down “command and control” style of leadership with decision making highly centralised with minimal delegation of authority to departments and front-line staff. Some interviewees described the style of leadership as “dictatorial” and “abrasive” and one “that did not place enough trust in staff”. It emerged that the senior leadership teams of both the low performing trusts rarely engaged with staff lower down the hierarchy or consulted the views of service users making key decisions. This lack of general engagement with staff adversely affected the results of internal staff surveys which in turn reflected external regulators (CQC) poor assessment of the organisation's leadership. It was reported however, that the low preforming trusts were beginning to think about developing more inclusive approaches to involving staff in decision-making. In contrast, in the two high performing trusts the style of board leadership was described as being devolved, “collaborative and inclusive”, with the board consulting and involving staff lower down the hierarchy in key strategic decisions and devolving more autonomy to directorates and frontline services. Here staff were encouraged to be actively involved in problem solving and innovation at all levels, to put forward suggestions and be actively involved in effecting positive change. This more inclusive style of leadership with the emphasis on ensuring that staff feel safe, supported, respected and valued at work, was reflected in more positive scores for staff engagement in local and national staff surveys across the high performing trusts compared with the low performing trusts.

A striking difference between high and low performing organisations related to the strategic priorities of the trust board. In the low performing trusts the approach to strategy formulation tended to be “ad hoc and fragmented”. Here the focus was on maintaining a financial balance and bearing down on costs, which in trust A for example had led to a low score on the CQC quality ratings. Whereas in the two high performing organisations the strategic priorities of the board had been clearly codified in document form (business plan and a well-articulated quality improvement strategy) which had been cascaded and embedded in accountability arrangements throughout the organisation. The focus was very much on ensuring and enhancing the quality and safety of services for patients and service users. And although financial discipline was viewed as important, cost containment and fiduciary priorities were viewed as subsidiary to ensuring the delivery of high-quality services.

### Organisation culture

Organisational culture is a contested concept but is generally taken to comprise that which is shared and taken for granted between members of an organisation. That might include, for example, the beliefs, values, codes of practice and social norms which guide working professional behaviour, as well as the routines, traditions ceremonies, taboos and rewards which underpin organisational life ( Mannion *et al* ., 2005b ). These shared ways of thinking and behaving help define what is legitimate and acceptable in a group organisational setting. In the English NHS, trust boards are tasked with the important role of creating, embedding and transmitting desirable values and standards of conduct for the organisations and its staff. Official documentation makes clear that trust boards are responsible for setting the tone for the entire organisation, not only through corporate communications but also through the alignment and consistency of board members' behaviours with the espoused culture promoted through organisational documents and initiatives. Over recent years and in the light of several high-profile scandals, which have demonstrated that uncaring and ineffective practices can flourish when the organisational context goes wrong, there has been an increasing drive to change the culture of NHS organisations so that they are more open, responsive and encouraging to frontline staff to speak up when things are array. The rationale being that supporting staff to be open about their mistakes and divulge instances of poor-quality care, allows valuable lessons to be learnt and remedial action instigated to prevent problems from recurring.

We found stark differences in the cultures of the high and low performing trusts in relation to how open they were about encouraging staff to voice concerns about poor quality care. In the two low performing trusts it was reported that staff at the apex of the organisation were less open to hearing and responding to concerns from staff lower down the hierarchy. As one interviewee from trust A commented, their trust was a “very closed off, top-down, non-listening organisation”. The two low performing organisations were reported to have “blame cultures” that were “quick to find fault” and discipline or “punish” staff members for reporting mistakes. The result was that because of the fear of recrimination staff were more reluctant and “fearful” of reporting incidents and raising legitimate concerns about poor quality care. The consequence is that they were unable to exert upward influence and the organisation did not benefit from learning from past mistakes with performance and quality problems unaddressed. Whereas in the high performing trusts it was reported that the leadership of each organisation generally encouraged more fair and open cultures in which staff felt more “psychologically safe” to raise concerns and were less likely to be “scapegoated” or suffer detriment for reporting concerns. This led to a general perception that staff lower down the hierarchy were listened to and their concerns were acted upon to prevent problems from recurring.

### Relationships in the local economy

Mental health trusts do not exist in isolation but are embedded within wider policy and organisational networks within their local health community and nationally. Leaders of mental health trusts therefore need to build and maintain successful relationships with other health and social care organisations whose active collaboration and cooperation are essential to the effective delivery of care services. We found some differences in the style and approach towards engaging local organisations and key external stakeholders between the two high and two low performing mental health trusts. The two low performing trusts were both characterised as having relatively poor or “challenged” relationships with local partner and commissioning organisations. For example, staff in trust A reported having a competitive and detached relationship with their local acute provider and staff in trust B reported strained relationships with the local authority and one of the acute providers in their area. This appears to be an artefact of the more “abrasive” approach to board leadership identified in the low performing trusts. In contrast, the two high performing trusts generally had much better relationships with local partner organisations in the wider health community, sharing ideas on quality and areas of concern for patients and service users. According to the medical director of trust D, while maintaining relationships was sometimes challenging in terms of the required investment in time and resources, the trust's leadership recognised the value of constructive engagement and the need for good relationships with key stakeholders and local health influencing organisations.

### Investment in technology and leveraging digital tools

New technology and in particular advances in telemedicine and digital interfaces have the potential to bring great benefits for improving the quality and efficiency of mental health services and to radically transform the way in which mental health care is delivered and accessed. This includes developments in remote, mobile and assistive technologies with the capacity for reduced administration, better diagnoses and treatments, as well as empowering patients and their carers to take on a more active role in self-managing their treatment. When it came to organisational investment in new technology and in particular the shift from analogue technology to digital platforms, we found that the two high performing trusts were much further ahead than the two low performing trusts and this was reflected in their degree of “digital maturity” and how enthusiastically they had embraced the potential for digital tools. This was further evidenced in the period following the interviews by both high performing organisations being presented with national awards in recognition of being leaders in digital technology through implementing a range of innovative organisation-wide digitally enabled systems such as paperless wards and services and digitising observations. This allowed both trusts to increase their investment in this area and further develop their technology infrastructure. In contrast, the two low performing trusts lagged behind in their technological infrastructure and digital capabilities with for example having to use outdated software programmes and incompatible computer systems because these had not been updated. This therefore limited their ability to harness the potential of digital technology for the benefit of patients and service users.

## Discussion

To the best of our knowledge this is the first study to systematically examine the relationship between organisational factors and the performance of mental health trusts. The evidence from our case studies suggests that high and low performing mental health trusts have a number of different and distinguishing organisational and management characteristics. Although each case possessed its own unique character, significant patternings were observed within cases grouped by performance to suggest a degree of divergence. To gain additional analytical purchase in interpreting our findings we present these against the background of the broader theoretical and empirical literature on management and organisational governance.

Board governance in the two low performing trusts was remarkably similar to the *Agency model of governance* which is based on the assumption that, unless scrutinised, staff will seek to pursue their own interests rather than wider organisational objectives (opportunism). Here the board is cast as a monitoring device set up to ensure compliance by developing organisational systems of checking, monitoring and control to hold staff accountable for their actions. The role of board leadership in this model can be best described as hierarchical, authoritarian and risk averse: setting direction and implementing mechanisms to be assured that the organisation follows. The downside of this approach to governance is that it can lead to a defensive culture where staff are fearful of reporting concerns and consequently opportunities are lost to act on this soft intelligence to improve performance ( Martin *et al* ., 2015 ).

In contrast the two high performing organisations exhibited characteristics which were much more aligned to the Stewardship model of board governance. This model works on the assumption that employees are motivated by more than their own narrow self-interests, and that staff want to do a good job and serve as effective stewards of an organisation's resources. The model assumes a high degree of trust on the part of senior leaders with an appetite for risk, with the focus of the board being keen on creating a framework for shared values and enabling staff, rather than monitoring and coercing performance. Leadership in this model is characterised as collective with organisational goals determined through inclusive dialogue and debate, with the aim of creating shared responsibility and cooperation across the organisation and with key stakeholders. The potential downside of this approach is that it can lead to Groupthink behaviour typical of highly cohesive groups which may inhibit the expression of (true) opinion; and in such cases group harmony and unanimity may be privileged over effective challenge to board members ( Mannion and Thompson, 2014 ).

Extending this analysis of board governance still further, Garratt (1997) has integrated the insights from both agency and stewardship theories and posits two main board leadership objectives, which he terms “conformance” and “performance” (see Table 3 ). *Conformance* has both external and internal dimensions: *external accountability* includes compliance with regulatory and legal requirements, as well as accountability to external stakeholders. In contrast the *internal dimension* is associated with management control. The *conformance dimension* therefore shares many similarities with the agency theory perspective on governance. The *performance dimension* , according to Garratt, involves governing the organisation to enhance its achievement of goals and objectives. This again consists of two main functions: *policy formulation* and *strategic thinking* . The performance dimension thus links closely to the stewardship theory of corporate governance. This framework (illustrated in Table 3 ) suggests that boards need to focus on both their conformance and performance aspects of corporate governance, and that blended perspectives on agency/stewardship may be necessary depending on external drivers and local context.

Consistent with this thinking, is our finding that although the two low performing trusts in our study were aligned closely with the agency model, they were beginning to see the limitations of this style of governance and starting to think about developing more devolved systems of governance, and evolving styles of governance, which were more aligned with stewardship approaches. As highlighted in Table 3 , both agency and stewardship approaches are important for beneficial organisational functioning, but no single approach to governance is likely to be the most appropriate for all contexts. Previous research indicates that the trick may be to mix and match the appropriate style to the stage in the performance cycle in which the organisation finds itself ( Mannion *et al* ., 2005a ). Thus, agency style approaches may be more useful in situations when an underperforming organisation requires strong central direction to establish robust internal performance management arrangements. Whereas high performing organisations with established performance management systems may benefit from developing more participatory and devolved styles of governance and other high trust management practices associated with stewardship approaches. What is clear is that in the governance of mental health providers there will definitely be trade-offs and a judicious mix of approaches and styles may be required to match changing external policy contexts with local service priorities and prevailing cultures.

A key difference identified in the case study work was that the high performing providers focussed on enhancing quality of services whereas the low performing organisations placed more of a strategic priority on containing costs. In theory, technical efficiency can be improved by either increasing quality or reducing costs (or a mixture of the two). However, empirical evidence suggests that in some contexts and service settings, improving quality can result in a virtuous cycle of cost reduction and may increase value for money across the whole health system ( Øvretveit, 2009 ). Whereas driving down costs can compromise quality and may ultimately lead to a vicious downward spiral with higher costs generated for the wider health system, some of which will be intangible and difficult to measure. Similarly, in some contexts, investment in technology has the potential to improve productivity and lower health care costs due to the design of work process and the adoption of less labour-intensive models of care, less time devoted to routine administrative tasks due to automation, a reduced need for travel and enhanced online support for patient engagement and self-care policies ( Black *et al* ., 2011 ; Imison *et al* ., 2016 ).

One of our key findings is regarding differences in organisational culture between high and low performing mental health trusts. Previous research has shown that leaders who create an environment of “psychological safety” that fosters assertive communication and a culture of open reporting encourage staff to bring forth concerns which creates opportunities for learning that can lead to overall organisational improvement ( Edmondson *et al* ., 2016 ). Yet any linkages between organisational culture and performance in mental health trusts are likely to be highly contingent, complex and nonlinear, making it an inherently difficult field to study ( Braithwaite *et al* ., 2017 ). One key difficulty (even with the use of in-depth qualitative approaches) lies in disentangling the direction of causality between internal factors such as organisational culture and performance. Although most attention to date centred on how culture may affect performance, it is equally plausible that certain cultures arise from high-performing organisations. That is, performance drives culture. More likely still is that culture and performance are recursive, mutually constituted and reinforcing. Indeed, the widely used phrase “the way things are done around here” could be interpreted as much a definition of performance as it is of culture ( Mannion *et al* ., 2005a ). Thus, simplistic ideas of “line up the cultural values” and high performance will follow can be seen as naïve.

Finally, we identified differences between high and low performing organisations with regard to the quality of their inter-organisational relationships in the local economy. Within the English NHS a range of possible inter-organisational collaboration types such as alliances, buddying, clinical networks and mergers exist with the potential to improve performance through reduced duplication of effort, enabling resource sharing and promoting shared learning and innovations ( Aunger *et al* ., 2021 ). And although previous research has identified good relationships within the local health economy as a marker of performance in NHS acute trusts ( Mannion *et al* ., 2005b ), there is currently a dearth of empirical research in this area to inform policy ( Aunger *et al* ., 2021 ).

## Limitations

As with all research projects it is important to acknowledge the limitations of the study when interpreting and applying the findings. The first relates to confidence in the generalisability of the qualitative findings beyond the four case study sites. Although the case studies were sampled purposefully to reflect organisations at either end of the performance continuum organisational characteristics, as well as being dispersed geographically across the country, we cannot state categorically that our findings are necessarily generalisable to all mental health trusts in England. However, we believe that our study informed by relevant theory, has uncovered some important insights that are transferable to an understanding of performance in mental health trusts more generally. A second limitation of this work may be in the focus on the perceptions of a small number of senior managers: we were unable to triangulate their perspectives and experiences with those of staff lower down the organisational hierarchy, including those working on the frontline. However, the benefit of focussing on senior staff is that they sit at the apex of an organisation and have a strategic overview of organisational strategies and organisational performance. We also interviewed managers from local commissioning groups to obtain an external perspective as well as patient representatives. Of course, the focus that we have chosen does not obviate the need for deeper study that explores how organisational process plays out at other layers of the organisation. A third limitation is that our study was cross-sectional and conducted at one point in time and therefore although we are able to identify associations between organisational processes and organisational performance, we are not able to prove causality. Nevertheless, this is a qualitative study and we believe that we have been able to disentangle some of the important organisational processes that drive performance which are difficult to identify through quantitative analysis alone. Finally, the sample of mental health organisations was drawn from the English NHS and our findings may not be generalisable to mental health providers in other countries with different health systems and user groups.

## Conclusions

How trust boards and senior managers in mental health trusts set direction, exercise control, shape culture, invest in technology and interact with partner organisations would appear to be associated with the performance of their organisation. In other words, our findings suggest that the approaches taken to govern and manage mental health trusts do matter, and our findings give clues as to what areas might need attention and where future action might be usefully directed. They include, but are not limited to: developing appropriate governance frameworks and organisational cultures, ensuring that staff across the organisation feel “psychologically safe” and able to speak up when they see things that are going wrong; a focus on enhancing quality of services rather than prioritising cost-reduction; investing in new technology and digital applications; and nurturing positive inter-organisational relationships across the local health economy. We would, nevertheless, consider that given the cross-sectional nature of the study that these insights are tentative and although we have found strong and plausible associations between particular organisational processes, management practices and organisational performance, we cannot establish causality or the direction of any causality. So, for example it is difficult to establish conclusively whether a particular leadership style is driving performance or is the outcome of having a high performing trust. As the phenomena of interest are essentially dynamic (performance and change), longitudinal study will offer important insights over cross-sectional designs and would be able to shed more light on the organisational drivers of performance. Despite such methodological reservations, our findings provide some indication that organisational processes and management practices may indeed matter in the delivery of high performance in mental health services.

## Figures and Tables

**Table 1:** Key informants by trust

Category	Trust A	Trust B	Trust C	Trust D
Board/executive level informants	1. Chair	1. Chief Executive	1. Medical Director	1. Chief Executive Officer
	2. Chief Executive	2. Finance Director	2. Director of Nursing	2. Medical Director
	3. Director of Finance and Information	3. Medical Director	3. Director of Finance and Performance	3. Director of Nursing
	4. Director of Nursing, AHP and Quality	4. Director of Operations	4. Chief Operating Officer	4. Chief Operating Officer
	5. Director of Strategy		5. Director of Strategy	5. Deputy CEO and Chief Finance Officer
	6. Medical Director		6. Director of Communications	
	7. Director of Operations			
Service level (management) informants	8. Associate Director of Operations	5. MH Head of Inpatient and Urgent Care	7. Clinical Manager CAMHS	6. Deputy Director of Nursing
	9. Head of Organisational Development, Leadership and Culture	6. Nurse Director – MH	8. Team Manager Older People Recovery	7. Regional Director West
	10. Associate Director of Operations – Old People's Division	7. MH and Learning Disability Service Director	9. CAMHS Manager	8. Regional Director East
	11. Clinical Lead IAPT	8. Lead Psychologist	10. Recovery Support Team Manager	9. Clinical Director, West
	12. Adult Community Lead	9. Personality Disorder Team Lead		10. Clinical Director for Adults MH Services
	13. Deputy Dir of Informatics	10. Early Intervention in Psychosis Lead		11. Service Manager EIP
		11. Mental Health Act Coordination Lead		
		12. Community MH Services Lead		
Consultant Psychiatrist	14. Consultant Psychiatrist Adult Service	13. Consultant Psychiatrist Child and Adolescent Services	11. Consultant Psychiatrist Adult Service	12. Consultant Child and Adolescent Services
Patient Rep and CCG informants	15. Patient Rep/Governor	14. Patient Rep	Non-response	13. Patient Rep 1
	16. CCG – Head of MH Commissioning	15. CCG – Executive Director		14. Patient Rep 2
	17. CCG – Lead for CAHMS	16. CCG Deputy Director of Commissioning		15. CCG_1
				16. CCG_2

**Table 2:** Divergence in the organisational and management practices between low and high performing mental health trusts

Organisational characteristics	Low performing MH trusts	High performing MH trusts
Board leadership style	Centralised command and control	Devolved
Board priorities	Cost control	Quality assurance/improvement
Staff engagement	Low	High
Trust in Staff	Low	High
Culture	Blame	Open
Relationship in local health economy	Poor	Good
Investment in technology and degree of digital maturity	Low	High

**Table 3:** The main functions of mental health trust board

	Short term focus on “conformance”	Long term focus on “performance”
External focus	*Accountability* Ensuring external accountabilities are met, e.g. to a range of stakeholders, funders, regulators Meeting external audit, inspection and reporting requirements	*Policy formulation* Stting and s transmitting the organisation's mission and values Long-term goal setting Ensuring appropriate organisational policies and systems are in place and adhered to
Internal focus	*Supervision* Recruiting, promoting and rewarding staff Managing performance processes Monitoring key financial and budgetary targets Managing quality safety and risks	*Strategic thinking* Agreeing strategic direction Shaping and agreeing long-term plans Reviewing and deciding on major resource decisions and investments

**Source(s):** Adapted and extended from Chambers *et al* . (2013)
